# Intravenous Heroin Induces Rapid Brain Hypoxia and Hyperglycemia that Precede Brain Metabolic Response

**DOI:** 10.1523/ENEURO.0151-17.2017

**Published:** 2017-06-07

**Authors:** Ernesto Solis, Keaton T. Cameron-Burr, Yavin Shaham, Eugene A. Kiyatkin

**Affiliations:** Behavioral Neuroscience Branch, National Institute on Drug Abuse, Intramural Research Program, National Institutes of Health, DHHS, Baltimore, MD 21224

**Keywords:** brain temperature, electrochemistry, opioids, rats, respiratory depression, vascular tone

## Abstract

Heroin use and overdose have increased in recent years as people transition from abusing prescription opiates to using the cheaper street drug. Despite a long history of research, many physiological effects of heroin and their underlying mechanisms remain unknown. Here, we used high-speed amperometry to examine the effects of intravenous heroin on oxygen and glucose levels in the nucleus accumbens (NAc) in freely-moving rats. Heroin within the dose range of human drug use and rat self-administration (100–200 μg/kg) induced a rapid, strong, but transient drop in NAc oxygen that was followed by a slower and more prolonged rise in glucose. Using oxygen recordings in the subcutaneous space, a densely-vascularized site with no metabolic activity, we confirmed that heroin-induced brain hypoxia results from decreased blood oxygen, presumably due to drug-induced respiratory depression. Respiratory depression and the associated rise in CO_2_ levels appear to drive tonic increases in NAc glucose via local vasodilation. Heroin-induced changes in oxygen and glucose were rapid and preceded the slow and prolonged increase in brain temperature and were independent of enhanced intra-brain heat production, an index of metabolic activation. A very high heroin dose (3.2 mg/kg), corresponding to doses used by experienced drug users in overdose conditions, caused strong and prolonged brain hypoxia and hyperglycemia coupled with robust initial hypothermia that preceded an extended hyperthermic response. Our data suggest heroin-induced respiratory depression as a trigger for brain hypoxia, which leads to hyperglycemia, both of which appear independent of subsequent changes in brain temperature and metabolic neural activity.

## Visual Abstract

**Figure:**
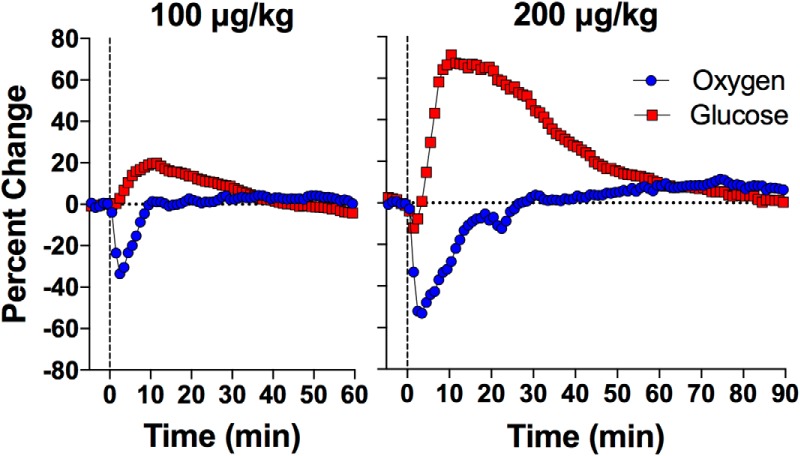

## Significance Statement

Heroin overdose has dramatically increased in recent years. Despite a long history of research, many physiological effects of heroin and their mechanisms remain unknown. We used high-speed amperometry to measure brain oxygen and glucose levels after intravenous heroin injections in freely-moving rats. We found that heroin induces a rapid drop in brain oxygen and established a role for drug-induced respiratory depression and decreases in blood oxygen in mediating this effect. Heroin-induced respiratory depression also results in increased CO_2_, which induces vasodilation and cerebral hyperglycemia. The rapid effects of heroin on brain oxygen and glucose are independent of slower changes in brain temperature and metabolic activity, challenging the prevailing dogma that metabolic demands trigger the intra-cerebral entry of oxygen and glucose.

## Introduction

Heroin is one of the best-known drugs of abuse and has increased in popularity during recent years as people using prescription opiates have switched to the cheaper and stronger illegal substitute (Compton et al., 2016). In addition to the acute subjective experience following intravenous administration or smoking, heroin induces multiple behavioral and physiological effects, including hypoactivity, sedation, inhibition of gastrointestinal activity, and analgesia (Jaffe et al., 1997; Simon, 1997; Baud, 2009). Like other opioid drugs, heroin induces respiratory depression, which, with increased dosage, can lead to hypoxia, comatose state, and lethality (Yeadon and Kitchen, 1989; White and Irvine, 1999). In recent years, there has been an alarming increase in the number of heroin-related deaths, and thus, it is necessary to better understand the physiological mechanisms of heroin action within the range of voluntary low to moderate drug intake and high dose drug intake that could result in overdose (Compton et al., 2016).

Many studies on heroin have focused on drug-induced changes in the activity of central neurons that mediate the drug’s reinforcing effects (Bozarth and Wise, 1981; Bozarth, 1994; Kiyatkin and Rebec, 2001; Robinson and Berridge, 2003; Kenny et al., 2006). However, neural activity and function are critically dependent on the proper inflow of oxygen and glucose from arterial blood into the brain tissue (Siesjö, 1978; Harris et al., 2012). Under physiological conditions, the entry of oxygen depends on the blood-brain concentration gradient and is modulated by neural activity via direct or indirect changes in vascular tone (vasoconstriction/vasodilation) and local cerebral blood flow (Attwell et al., 2010; Lecrux and Hamel, 2011). However, heroin and its active metabolites affect the activity of multiple central neurons, alter the tone of central and peripheral blood vessels, and induce respiratory depression, leading to a decrease in blood oxygen levels (Jaffe et al., 1997; Stohler et al., 1999). Therefore, heroin could significantly alter the entry of oxygen and other key metabolic substrates, such as glucose, into the brain. Here, we used high-speed amperometry with oxygen and glucose sensors in awake, freely-moving rats to study heroin’s effects on brain oxygen and glucose levels and their relationship to the drug’s effects on brain temperature and metabolism.

First, we examined the effect of low doses of intravenous heroin on extracellular levels of oxygen and glucose in the nucleus accumbens (NAc), a brain region that plays a critical role in heroin self-administration and relapse to heroin seeking (Wise and Bozarth, 1987; Di Chiara, 2002; Badiani et al., 2011). Second, we examined the relationships between heroin-induced changes in NAc glucose and oxygen levels and conducted oxygen and glucose recordings in the subcutaneous space to clarify mechanisms underlying heroin-induced changes in brain glucose and oxygen. Third, we examined whether heroin-induced changes in brain oxygen and glucose are related to changes in brain temperature, intra-brain heat production, and skin vascular tone, basic physiological parameters reflecting brain metabolic activity and heat loss to the external environment (Kiyatkin, 2010; Lenoir et al., 2013). Finally, we examined the effects of a very high heroin dose (an overdose condition) on oxygen and glucose levels in the NAc, as well as the above temperature parameters.

## Materials and Methods

### Subjects

Forty adult male Long-Evans rats (Charles River Laboratories) weighing 460 ± 40 g at the time of surgery were used in this study. Rats were individually housed in a climate-controlled animal colony maintained on a 12/12 h light/dark cycle (lights on at 6 A.M.), with food and water freely available. All procedures were approved by the NIDA-IRP Animal Care and Use Committee and complied with the Guide for the Care and Use of Laboratory Animals (NIH, Publication 865-23). Maximal care was taken to minimize the number of experimental animals and any possible discomfort or suffering at all stages of the study.

### Overview of the study

Our study describes the results of five electrochemical experiments conducted in awake, freely-moving rats. In two experiments, we examined the effects of intravenous heroin on extracellular levels of oxygen (experiment 1) and glucose (experiment 2) in the NAc. To clarify possible mechanisms underlying heroin-induced changes in NAc oxygen and glucose, in two experiments we examined the effect of intravenous heroin on oxygen (experiment 3) and glucose (experiment 4) levels in the subcutaneous space. To explore the relationship between changes in oxygen, glucose, and metabolic brain activation, we analyzed oxygen and glucose data in conjunction with data from a thermorecording experiment (experiment 5), in which we assessed heroin-induced temperature changes recorded in the NAc, temporal muscle, and skin. In experiment 6 we examined the effects of heroin at a very high dose (3.2 mg/kg) within the range of presumed overdose, on NAc oxygen, glucose, and multiple temperature parameters.

### Surgical preparations

Surgical procedures for electrochemical experiments have been described in detail elsewhere (Kiyatkin and Lenoir, 2012; Wakabayashi and Kiyatkin, 2012). In brief, under general anesthesia (Equithesin, a mixture of sodium pentobarbital and chloral hydrate), each rat was unilaterally implanted with a BASi guide cannula (Bioanalytical Systems) into which an electrochemical sensor was later inserted. In experiments 1, 2, and 5, cannulas were implanted for recordings in the right NAc shell. Target coordinates of the recordings were: AP +1.2 mm, ML ±0.8 mm, and DV +7.6 mm from the skull surface, according to coordinates of the rat brain atlas (Paxinos and Watson, 1998). In experiments 3 and 4, cannulas were implanted subcutaneously in the medio-frontal area of the rat’s head. The guide cannula was secured with dental acrylic in a head mount anchored to the skull. When not in use, stainless steel obdurators were inserted into the cannulas to prevent occlusion. During the same surgical procedure, rats were also implanted with a chronic jugular catheter, which ran subcutaneously to the head mount and was secured to the same head assembly. Rats were allowed a minimum of 5 d of postoperative recovery and at least three daily habituation sessions (∼6 h each) to the recording environment; jugular catheters were flushed daily with 0.2 ml of heparinized saline to maintain patency.

Surgical procedures for thermorecording experiments have been described in detail elsewhere (Kiyatkin et al., 2013). In brief, under the same general anesthesia protocol, rats were implanted with a jugular catheter and three copper-constantan thermocouple electrodes in the NAc shell (AP +0.8–1.2 mm, ML +/−0.8 mm, and DV +7.4 mm from the skull surface), temporal muscle, and subcutaneously along the nasal ridge with the tip ∼15 mm anterior to bregma. The probes were secured with dental cement to three stainless steel screws threaded into the skull. As described in our previous studies (see Kiyatkin, 2010; Kiyatkin et al., 2014 for review), by simultaneously recording temperature from these three locations, it is possible to assess the effects of physiological or drug stimuli on intra-brain heat production due to metabolic brain activation and heat loss or retention due to changes in skin vascular tone (vasoconstriction/vasodilation). Specifically, the temperature difference between brain and temporal muscle (the NAc-muscle differential), which both receive arterial blood supply from the carotid artery, allowed us to evaluate the source of heat production, and thus isolate the effect of heroin on brain metabolism. The temperature difference between skin and temporal muscle (the skin-muscle differential) is a reliable index of peripheral vascular tone (i.e., vasoconstriction/vasodilation), a critical factor affecting heat accumulation in and loss from the brain (Kiyatkin, 2010; Kiyatkin et al., 2014). In these experiments, we also measured heroin-induced changes in locomotor activity using four infrared motion detectors (Med Associates), as previously described (Brown and Kiyatkin, 2004).

### Electrochemical detection of oxygen and glucose

In experiments assessing oxygen, we used commercially produced oxygen sensors (Model 7002-02; Pinnacle Technology). These sensors consist of an epoxy-sheathed disk electrode that is grounded to a fine surface using a diamond-lapping disk and are prepared from Pt-Ir wire 180 μm in diameter, with a sensing area of 0.025 mm^2^ at the sensor’s tip. The active electrode is incorporated with an integrated Ag/AgCl reference electrode. Dissolved oxygen is reduced on the active surface of these sensors, which is held at a stable potential of −0.6 V versus the reference electrode, producing an amperometric current. The current from the sensor is relayed to a computer via a potentiostat (Model 3104, Pinnacle Technology) and recorded at 1-s intervals, using the PAL software utility (version 1.5.0, Pinnacle Technology).

Oxygen sensors were calibrated at 37°C by the manufacturer (Pinnacle Technology) according to a standard protocol described elsewhere (Bolger et al., 2011). The sensors produced incremental current rises with increases in oxygen concentration within the wide range of previously reported brain oxygen concentrations (0–50 μM). Substrate sensitivity of oxygen sensors varied from 1.03–1.8 nA/1 μM (mean = 1.39 nA/1 μM). Oxygen sensors were also tested by the manufacturer for their selectivity toward other electroactive substances, including dopamine (0.4 μM) and ascorbate (250 μM), none of which had significant effects on reduction currents.

For monitoring brain glucose, we used commercially produced glucose oxidase-based biosensors (Model 7002; Pinnacle Technology). These sensors are prepared from Pt-Ir wire of 180 µm in diameter, with a sensing cavity of ∼1 mm in length on its tip. The active electrode is incorporated with an integrated Ag/AgCl reference electrode. On the active surface, glucose oxidase converts glucose to glucono-1,5-lactone and hydrogen peroxide (H_2_O_2_), which is detected as an amperometric oxidation current generated by a +0.6 V applied potential (Hu and Wilson, 1997). The potential contribution of ascorbic acid to the measured glucose currents is competitively reduced by co-localizing ascorbic acid oxidase on the active surface of the sensor (Hu and Wilson, 1997). This enzyme converts ascorbic acid to nonelectroactive dehydroascorbate and water. In addition, a negatively charged Nafion polymer layer under the enzyme layer serves to exclude endogenous anionic compounds (Hu and Wilson, 1997).

Glucose sensors were calibrated immediately before and after each *in vivo* experiment (Solis et al., 2017). *In vitro* calibrations were conducted in PBS, pH 7.3, by incrementally increasing the concentration of glucose (Sigma-Aldrich) from 0 to 0.5, 1.0, and 1.5 mM followed by a single addition of ascorbate (250 µM). Within this physiological range of glucose levels (Fellows and Boutelle, 1993; McNay et al., 2001), glucose sensors used in this study produced incremental linear current increases. Mean sensitivity to glucose was 4.79 ± 0.35 nA/0.5 nM at 22–23°C and 9.39 ± 0.68 nA at 37°C. Glucose sensors showed low sensitivity to ascorbate (0.04 ± 0.01 nA/250 µM at 22–23°C) and, as shown previously, were only minimally sensitive to dopamine at its physiological levels (5–50 pA/10–100 nM). Glucose sensors remained equally sensitive to glucose and selective against ascorbate during postrecording *in vitro* calibrations.

### Experimental procedures

All *in vivo* electrochemical procedures were performed in an electrically insulated chamber (38 × 47 × 47 cm) located inside a larger open-faced cabinet. The cage was illuminated continuously with a dim 20-W light bulb. Ambient temperature was maintained within 22–23°C and a room wide air filter fan provided background noise. The bottom of the cage was covered with wood chip bedding, which remained in place during the habituation and recording sessions for each individual rat.

At the beginning of each experimental session, rats were minimally anesthetized (<2 min) with isoflurane and the sensor was inserted either into the NAc (experiments 1, 2, and 5) or subcutaneous space (experiments 3 and 4) through the guide cannula. The rat was then placed in the testing chamber and the sensor was connected to the potentiostat via an electrically shielded flexible cable and a multi-channel electrical swivel. The injection port of the jugular catheter on the head mount was connected to a plastic catheter extension that allowed stress-free drug delivery from outside the cage, thus minimizing possible detection of iv drug injections by the rat. Testing began a minimum of 120 min after insertion of the sensor when the baseline currents stabilized.

In the first two experiments (*n* = 17), we examined changes in oxygen and glucose induced in the NAc by intravenous heroin (diamorphine HCl; obtained from the NIDA-IRP Pharmacy) at 100 and 200 μg/kg doses (in 0.2 and 0.4 ml of saline over 20 and 40 s) over the course of 6- to 8-h recording sessions. These doses were chosen as typical (100 μg/kg) or twice the typical unit dose that has been used in many heroin self-administration studies on mechanisms of heroin reward and relapse (Wise, 1989; Bossert et al., 2013). Time intervals between injections were 90–120 min, typically enough for restoration of preinjection current baselines. To investigate the mechanisms underlying the observed changes in NAc oxygen and glucose, the effects of heroin at the same doses were also examined in the subcutaneous space, a densely-vascularized area with little or no metabolic activity (experiments 3 and 4). In experiments with glucose monitoring (experiment 4), rats also received a single injection of 10% glucose (60 mg in 0.6 ml saline delivered over 40 s). Given that “normal” blood glucose levels in adult rats are within 5.4–7.0 mM (Smith and Pogson, 1977) and that blood volume is ∼30 mL, we estimate that intravenous delivery of 60 mg of glucose may maximally triple blood glucose levels (12 mM increases) when distributed within the entire blood volume. Increases in the glucose levels in the subcutaneous space following glucose injections were used to verify the subcutaneous location as an area that accurately represents changes in glucose levels in the arterial blood.

The experimental protocol for the thermorecording experiments (experiment 5) was similar to that used in the electrochemical experiments, with some minor changes due to multi-session recordings. Rats (*n* = 6) received three intravenous injections of 100 μg/kg heroin during the first treatment session and one intravenous injection of heroin at a double dose (200 μg/kg) during the second treatment session conducted after one drug-free day. In four different rats, we examined the temperature effects of a very high intravenous heroin dose (3.2 mg/kg).

In five additional rats (experiment 6), we examined the effects of heroin delivered at a low dose (100 μg/kg) followed by a very high dose (3.2 mg/kg, within overdose range). At the end of each session, the rats were anesthetized with Equithesin, disconnected from the potentiostat, and the biosensor was carefully removed and recalibrated. Typically, rats underwent only one recording session and on completion they were euthanized by decapitation under deep isoflurane anesthesia. Then, the brain was removed, stored in 10% formalin, and sectioned for verification of sensor placement using a rat stereotaxic atlas (Paxinos and Watson, 1998); the brain sections were also assessed for possible gross anatomic damage. In several rats, electrochemical recordings were repeated two to 4 d later.

### Data analysis

Electrochemical data were sampled at 1 Hz (i.e., mean current over 1 s) using the PAL software utility (version 1.5.0, Pinnacle Technology) and analyzed with two different time resolutions. Slow changes in electrochemical current were analyzed with 60-s quantification bins for 10 min before and 90 min after heroin injection. Rapid current changes were analyzed with 4-s bins for 40 s before and 300 s after stimulus onset. Although data were sampled with 1-s temporal resolution, the 4-s bin appears to be optimal for detecting rapid current changes within the time scale of our stimuli while simultaneously reducing the contribution of electrical noise.

Electrochemical data were first analyzed as raw currents. Because each of the individual sensors differed slightly in background currents and substrate sensitivity *in vitro*, currents were then transformed into concentrations and represented as relative concentration changes, taking a prestimulus baseline current as 0. Basal oxygen currents and their concentration equivalent were also used to estimate basal levels of oxygen in the NAc and subcutaneous space. One-way repeated measure ANOVAs (followed by Fisher PLSD *post hoc* tests) were used to evaluate the statistical significance of drug-induced changes in oxygen and glucose concentrations. The latency of oxygen response was determined based on the first data point significantly different from baseline (*p* < 0.05, Fisher test). To assess the magnitude of heroin-induced changes in oxygen and glucose, data were additionally analyzed as percentage change from baseline, where the baseline is represented as 0% deviation. We also used correlation analyses to examine the relationship between oxygen, glucose, and several temperature parameters.

## Results

### Effect of low doses of heroin on NAc oxygen and glucose levels

#### Oxygen

**Figure 1.**
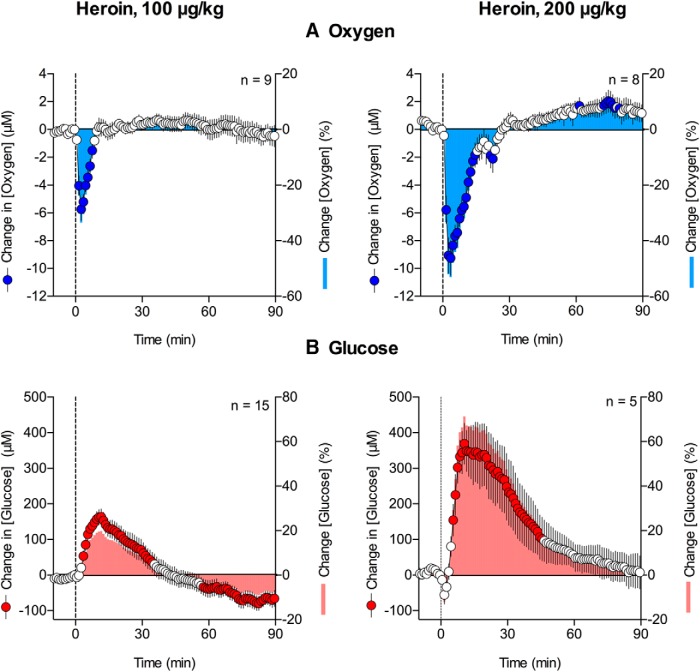
Relative changes in NAc oxygen (***A***) and glucose (***B***) induced by intravenous heroin at 100 and 200 μg/kg doses. Circles represent 1-min average (±SEM) changes calibrated in μM. Bars represent percentage changes with respect to baseline concentrations. *n* = number of averaged responses. Filled symbols show values significantly different from preinjection baseline. Vertical hatched lines show the onset of heroin injection.

**Figure 2.**
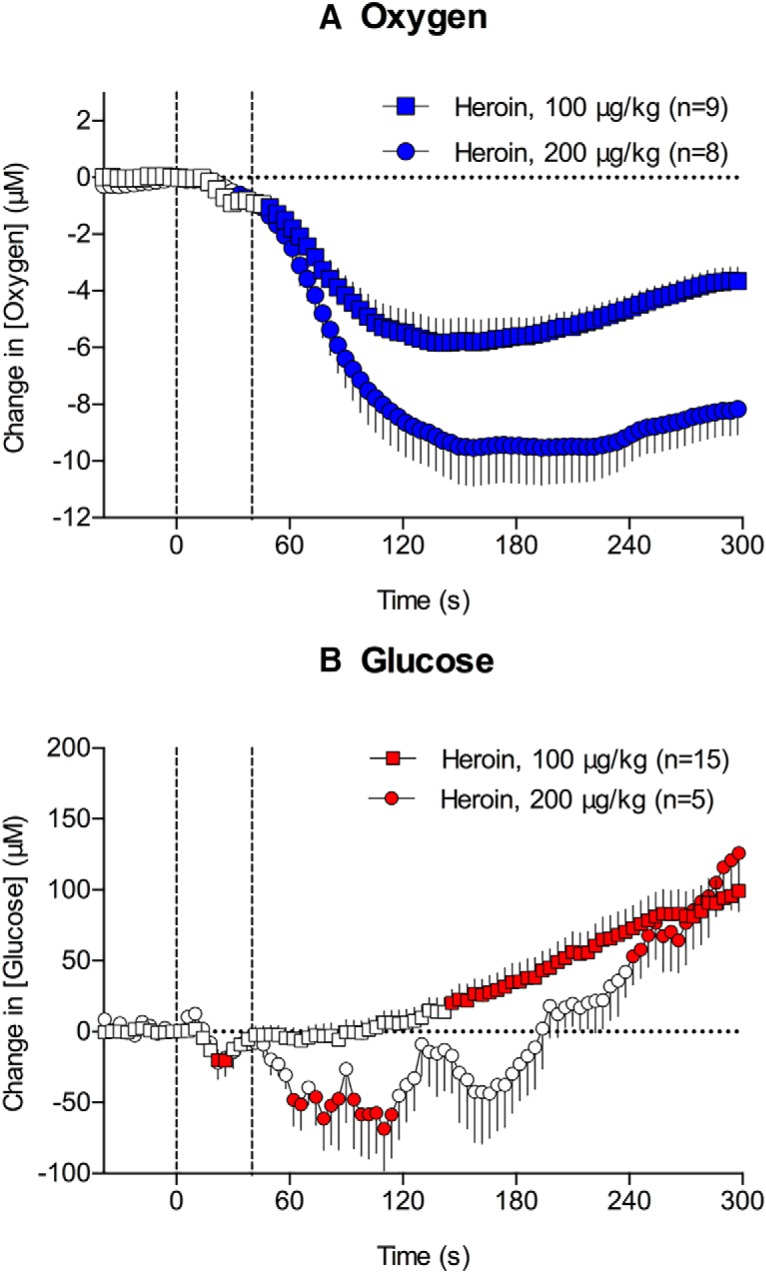
Rapid changes in NAc oxygen (***A***) and glucose (***B***) induced by intravenous heroin at 100 and 200 μg/kg doses. Circles represent 4-s mean (±SEM) changes calibrated in μM. *n* = number of averaged responses. Filled symbols show values significantly different from preinjection baseline. Vertical hatched lines show the onset and offset of heroin injection.

Intravenous heroin (100 μg/kg) administered to freely-moving rats under quiet resting conditions induced a significant decrease in NAc oxygen levels (*F*_(8,720)_ = 8.6, *p* < 0.001). When analyzed with slow (1-min) time resolution, the increase became significant during the second minute after the injection onset, reached nadir at 3–4 min, and disappeared ∼10 min after injection (Fig. 1*A*). When analyzed with rapid (4-s) temporal resolution (Fig. 2*A*), the decrease was significant (*F*_(8,600)_ = 25.5, *p* < 0.0001), had ∼44-s latency, and reached nadir at ∼145 s after the injection onset. Considering 17.2 μM as our estimate of NAc basal oxygen levels, the mean heroin-induced oxygen decrease at this dose was ∼33% below baseline. The observed decrease in oxygen became larger and more prolonged when the dose of heroin was doubled (200 μg/kg; *F*_(7,630)_ = 23.3, *p* < 0.001; Fig. 1*A*). In this case, the mean decrease was ∼9.3 μM or ∼54% below baseline. The decrease became significant during the second minute postinjection and reached nadir at 3–4 min. However, the return to baseline was more prolonged when compared with the rebound in oxygen following administration of 100 μg/kg of heroin (∼20 vs ∼10 min). Rapid time course analyses (Fig. 2*A*) showed that the decrease in NAc oxygen levels was significant (*F*_(7,525)_ = 50.7, *p* < 0.001) and had ∼50-s latency which is similar to that seen with the 100 μg/kg dose. Its nadir, however, occurred much later, ∼180–200 s after injection.

#### Glucose

Heroin significantly increased NAc glucose levels in a dose-dependent manner (Fig. 1*B*; *F*_(14,1260)_ = 37.8 and *F*_(4,360)_ = 13.9 for 100 and 200 μg/kg, respectively, *p* < 0.001). Compared with the oxygen decrease, the increase in glucose was delayed (∼4 and 6 min for 100 and 200 μg/kg, respectively) and much larger in absolute magnitude though similar in mean percentage deviation from baseline levels (∼170 and 350 μM, respectively, or ∼21 and 48% above baseline, which was ∼765 μM). Furthermore, glucose decreased transiently for 2–3 min immediately following the injection of the higher heroin dose (200 μg/kg; Fig. 1*B*). This qualitative change was more evident when the data were analyzed with high temporal resolution (*F*_(14,1050)_ = 27.0 and *F*_(4,300)_ = 9.1, 100 and 200 μg/kg, respectively, *p* < 0.001; Fig. 2*B*). The decrease was evident for the 200 μg/kg dose during the initial 3 min after the injection onset, which was followed by the rapid increase in glucose levels. A transient, weak drop in glucose was also seen when rats received the 100 μg/kg dose; however, this effect was not evident when data were analyzed with low temporal resolution.

Since basal levels of both oxygen and glucose varied between individual injections and were very different quantitatively [oxygen: 17.2 μM (SD = 8.98 μM) *n* = 17; glucose: 765.94 μM (SD = 246.18 μM) *n* = 20], we normalized each data set and calculated the percentage change of the mean from basal values. Basal levels were calculated as an average of mean values during the 3 min before injection and are represented as 0% change (Fig. 1). Using this type of analysis, we found that the mean magnitudes of the oxygen and glucose responses were relatively similar though opposite in direction (i.e., 20–30% with a lower heroin dose and 50–70% with a higher dose). For both doses, heroin-induced changes in oxygen were more rapid and more transient than changes in glucose.

### Relationship between heroin-induced changes in oxygen and glucose

**Figure 3.**
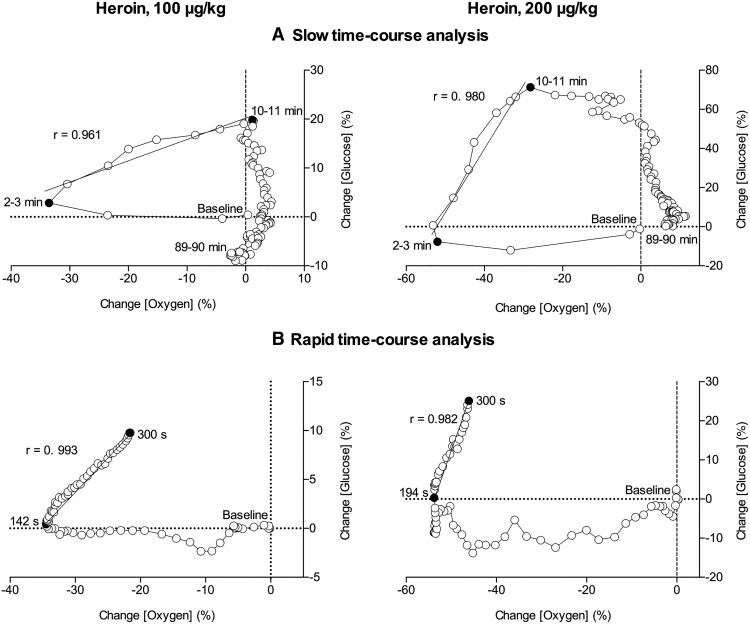
Correlative relationships between slow (***A***, 1-min bin) and rapid (***B***, 4-s bin) changes in oxygen and glucose induced by iv heroin at 100 and 200 μg/kg doses. Initial value immediately preceding heroin injection (0 for both oxygen and glucose) is marked as baseline. The period assessed for correlation is shown as the period between filled black circles. A line of best fit is shown with the correlation coefficient (*r*). Data are shown as percentage change from baseline. For details, see Results.

Both oxygen and glucose continuously enter the brain tissue from arterial blood via a gradient-dependent mechanism. The fact that both substances rely on gradient-dependent diffusion suggests that their drug-induced changes in the brain might be intertwined. To investigate this potential relationship, we used correlation analyses with respect to time after heroin injection. Figure 3 shows the results of these analyses conducted for slow and rapid changes of these substances.

As shown in Figure 3*A*, during the first 3 min following the injection of the low dose (100 μg/kg), we observed a rapid and strong oxygen decrease and no changes in glucose (no correlation). However, our data show an inflection point in the third minute postinjection, when oxygen begins to return to baseline and glucose begins to increase. We see a strong correlation (*r* = 0.961, *p* < 0.001) across this time interval when oxygen and glucose levels are both increasing (3–10 min). After 10 min, oxygen levels remain stable while glucose returns to baseline levels. A similarly strong correlation is observed when data are analyzed at a rapid time course with *r* = 0.993 (*p* < 0.001) between 142 and 300 s after injection (Fig. 3*B*).

Glucose and oxygen have similar dynamics when the dose of heroin is doubled to 200 μg/kg. As shown in Figure 3*A*, during the first 3 min after injection we see a rapid and robust decrease in oxygen. An inflection point appears in the third minute postinjection when oxygen begins to return to baseline and glucose levels begin to increase. The correlation is strong between 3–10 min after injection (*r* = 0.980, *p* < 0.001). The rapid time course dynamics are also similar to the low heroin dose, and though we observe a small delay in the oxygen rebound (194–300 s), the correlation between glucose and oxygen during this time interval was very high (Fig. 3*B*; *r* = 0.982, *p* < 0.001). In contrast to 100 μg/kg, we observed a small decrease in glucose levels during the first 3 min after injection when oxygen levels are decreasing. This effect is particularly evident in the rapid time course analysis. The return to baseline for oxygen was prolonged at the higher dose, as is evident in Figure 1. The glucose response appears to level out at ∼70% increase from the mean, which likely demonstrates maximum kinetics of glucose entry into the brain for this dose.

### Mechanisms underlying opposing changes in NAc oxygen and glucose induced by heroin

The data shown above suggest that heroin-induced decreases in NAc oxygen may result from decreased oxygen levels in arterial blood. In contrast, since glucose levels in arterial blood are strictly regulated and maintained at highly stable levels (Gerich, 1993), increases in NAc glucose induced by heroin could result from local vasodilation that enhances intra-brain glucose entry from arterial blood but not from a rise in blood glucose levels. To test these two possibilities and to further clarify the relationship between heroin-induced hypoxia and hyperglycemia, we conducted two electrochemical experiments in which we monitored oxygen and glucose in the subcutaneous space, a highly vascularized area with no or minimal metabolic activity.

**Figure 4.**
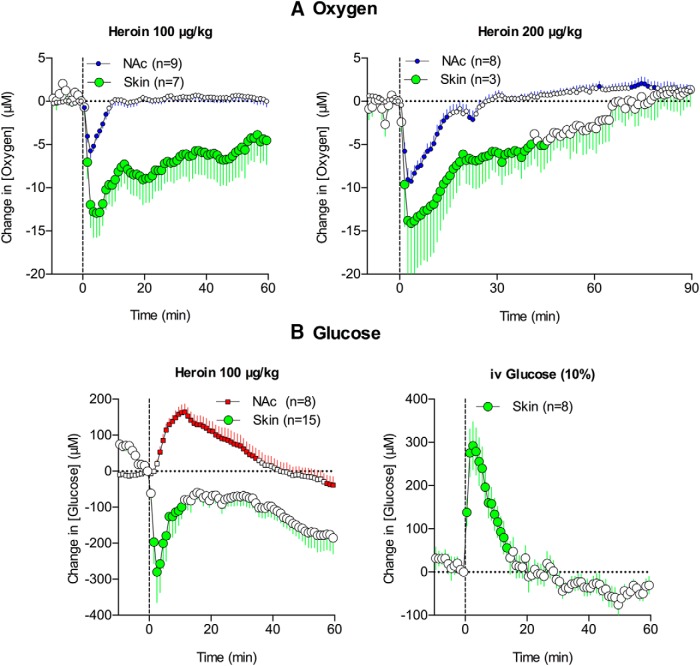
Mean (±SEM) changes in oxygen in the subcutaneous space (***A***) induced by heroin at 100 and 200 μg/kg doses. Changes in glucose concentration in the subcutaneous space (***B***) induced by iv injection of 100 μg/kg heroin and 10% glucose solution. Graphs of heroin injections also show data obtained in the NAc for comparison. *n* = number of averaged responses. Filled symbols indicate values significantly different from the preinjection baseline. Vertical hatched lines show the onset of heroin injection.

As shown in Figure 4*A*, heroin (100 and 200 μg/kg) significantly decreased oxygen levels in the subcutaneous space (*F*_(6,689)_ = 6.7 and *F*_(5,643)_ = 5.6, *p* < 0.0001); this effect was larger and more prolonged than the effect seen in the NAc. In contrast to the NAc where the oxygen decrease was transient, the oxygen decrease in the subcutaneous space was much longer in duration, and oxygen levels modestly decreased following subsequent heroin injections.

**Figure 5.**
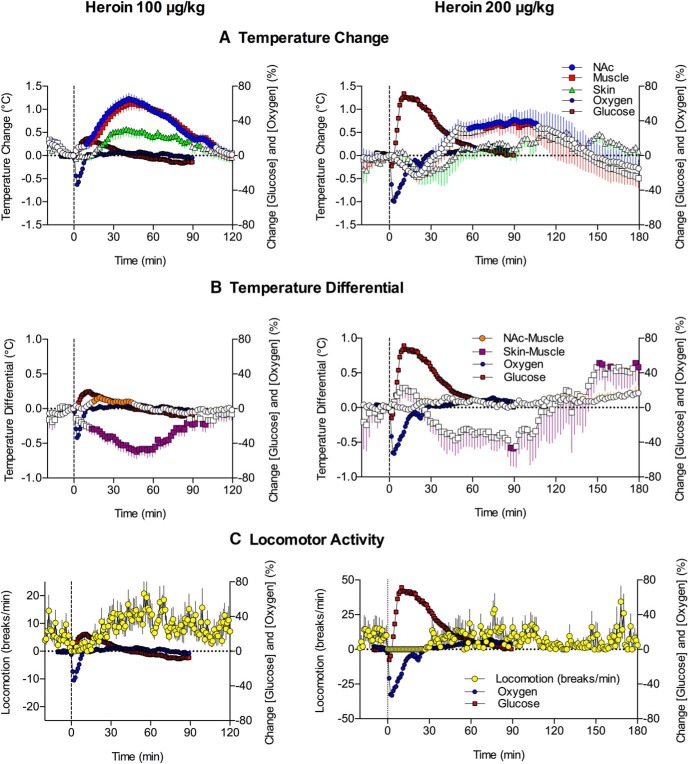
Mean (±SEM) changes in several temperature parameters (***A***, temperatures recorded from the NAc, temporal muscle, and skin; ***B***, NAc-muscle and skin-muscle temperature differentials) and locomotion (***C***) induced by heroin at 100 and 200 μg/kg doses. Percentage changes in NAc oxygen and glucose are superimposed on each graph. Filled symbols for temperature parameters show values significantly different from preinjection baseline. Data for oxygen and glucose are shown without standard errors for clarity.

Subcutaneous glucose levels increased rapidly and strongly when rats were injected intravenously with 10% glucose (*F*_(7,140)_ = 24.64, *p* < 0.001; Fig. 4*B*). This effect was comparable to, but slightly more phasic than, a previously reported rise in NAc glucose after an intravenous glucose injection at the same dose (Wakabayashi et al., 2016), suggesting that glucose levels in both the brain and subcutaneous space passively increase following the rapid rise in blood glucose levels. In contrast to the NAc, where glucose levels increased following heroin injection, heroin induced a significant decrease in glucose levels at the subcutaneous site (*F*_(7,154)_ = 4.9, *p* < 0.001; Fig. 4*B*). This decrease likely occurs due to skin vasoconstriction that diminishes glucose entry from blood vessels into the subcutaneous space. This decrease was rapid and prolonged, matching the vasoconstriction seen in this location following intravenous heroin injections (See Fig. 5 below).

### Effect of heroin on temperature and its relationship to heroin-induced changes in NAc oxygen and glucose

As shown in Figure 5*A*, heroin (100 μg/kg) increased brain, muscle, and skin temperature (*F*_(17,1037)_ = 26.4, *F*_(17,1037)_ = 21.7, and *F*_(17,1037)_ = 8.1, respectively; *p* < 0.001). The increases in NAc and muscle temperature were generally parallel, but the NAc-muscle temperature differential significantly increased from 10–20 min after injection (*F*_(17,1037)_ = 5.3, *p* < 0.001; Fig. 5*B*), suggesting intra-brain heat production due to metabolic brain activation. Skin temperature depends on two factors, the tone of blood vessels and the temperature of arterial blood inflow. Skin-muscle temperature differential, or skin minus muscle temperature, excludes the latter influence. This is because both skin and muscle receive the same temperature influence of arterial blood. Therefore, the differential reveals the effect of the drug on vascular tone. Due to the different time course of temperature changes in the skin and temporal muscle, this parameter strongly and monophasically decreased (*F*_(17,1037)_ = 7.8, *p* < 0.001), suggesting peripheral vasoconstriction. Heroin also induced a transient decrease in locomotor activity (freezing), which was evident within the first 15–20 min after injection (Fig. 5*C*).

As shown in Figure 5*A*,*B*, the heroin-induced drop in NAc oxygen occurred before any changes in temperature parameters and oxygen levels returned to baseline just as brain temperature began to increase. There was no correlation between oxygen and any temperature parameter (*r* = −0.155, *r* = −0.286, and *r* = −0.158 for brain temperature, NAc-muscle differential, and skin-muscle differential, respectively). While more delayed and prolonged, heroin-induced elevations in NAc glucose also showed no correlation with any temperature parameter and returned to baseline when the brain temperature peaked. However, heroin-induced motor inhibition was largely confined to the period in which oxygen dropped and glucose showed maximal acceleration (Fig. 5*C*).

Increases in brain and muscle temperature were also observed with the 200 μg/kg heroin dose (*F*_(4,364)_ = 3.2 and *F*_(4,364)_ = 3.3, respectively, *p* < 0.001; Fig. 5*A*). Though these increases were more prolonged than those observed with the 100 μg/kg dose, they were smaller in magnitude and preceded by temperature decreases within 10 min after heroin injections. The 200 μg/kg heroin dose did not change the NAc-muscle differential but induced two-phasic, up-down changes in skin-muscle differential (*F*_(4,484)_ = 2.8, *p* < 0.001), suggesting initial skin vasodilation followed by stronger and more prolonged vasoconstriction (Fig. 5*B*). Heroin-induced freezing also became stronger and more prolonged with the 200 μg/kg dose (Fig. 5*C*). While the decreases in oxygen were larger after the 200 μg/kg dose, they did not correlate with any temperature parameters. In contrast, a significant correlation was found between the increases in glucose occurring within a 60-min interval and both the decrease in brain temperature (*r* = −0.766, *p* < 0.001) and decrease in skin-muscle differential (*r* = 0.643, *p* < 0.001). Thus, when NAc glucose levels began to decrease from their peak, brain temperature began to increase from the dip observed after heroin injections. Although the glucose rise was more rapid than the increase in skin-muscle differentials, a significant correlation between these parameters resulted mainly from the parallel decreases in both parameters after their postinjection peaks. Like the 100 μg/kg heroin dose, the period of motor inhibition induced by the 200 μg/kg dose corresponded to the duration of the oxygen decrease and most of the glucose increase (Fig. 5*C*).

### Effects of a high heroin dose on NAc oxygen and glucose

**Figure 6.**
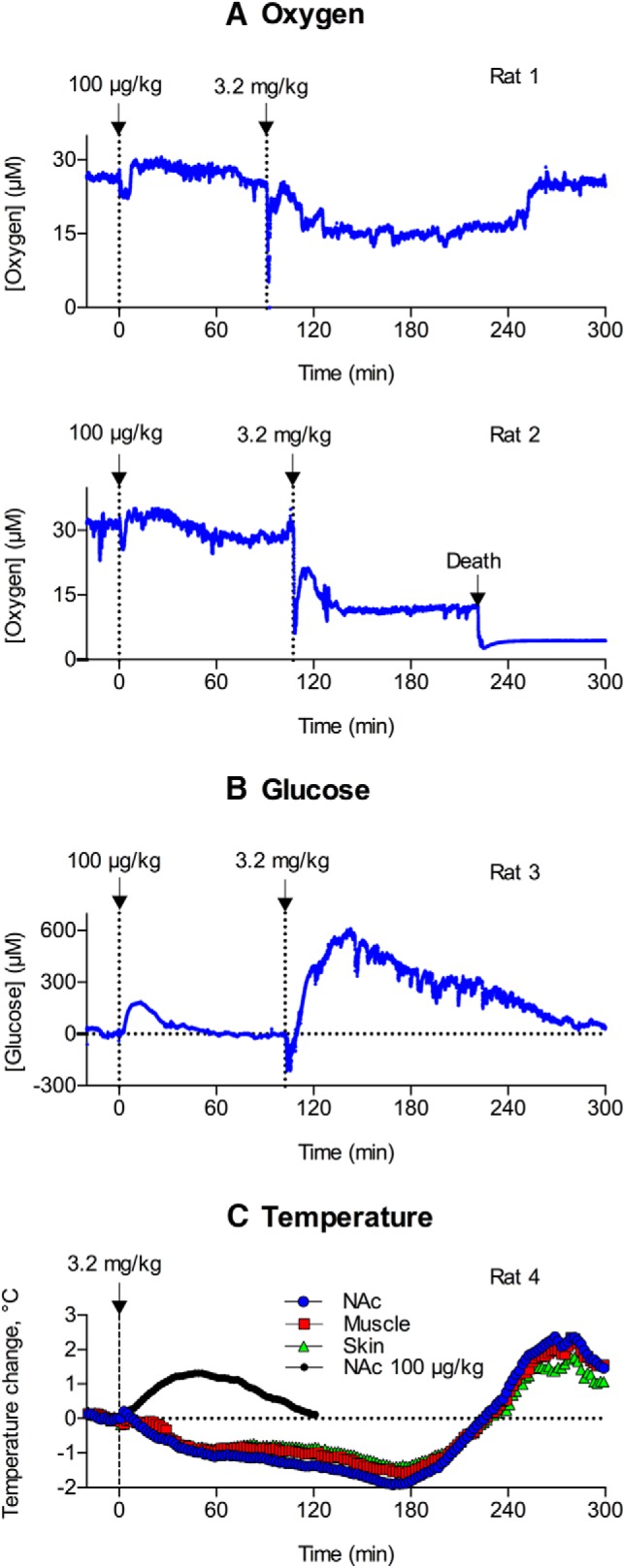
Original examples of changes in oxygen (***A***) and glucose (***B***) currents (1-s bins) following intravenous injections of heroin at a large dose (3.2 mg/kg). Each electrochemical record also shows changes induced by heroin at 100 μg/kg dose. C shows changes in NAc, muscle, and skin temperature induced by heroin at the same large dose. Small black circles on C show mean NAc temperature response induced by heroin at 100 μg/kg dose.

It is known that habitual heroin users develop tolerance to the positive psychoactive effects of the drug (Jaffe et al., 1997). To compensate, individuals may consume heroin at dramatically high doses, which may pose significant health risks. As such, there is clinical interest in what happens physiologically when heroin is administered at very high doses. To address this issue, we conducted several electrochemical recordings in rats, which were injected with a 3.2 mg/kg heroin dose that is 32-fold larger than the frequently self-administered dose used in our study (100 µg/kg). Single-case examples of glucose, oxygen, and temperature responses induced by heroin at this large heroin dose are shown in Figure 6. Responses to the “standard” 100 μg/kg heroin dose are shown for comparison.

The high heroin dose (3.2 mg/kg) induced a biphasic and prolonged decrease in NAc oxygen (Fig. 6*A*). Oxygen levels dropped dramatically (80% decrease) immediately after the injection and then rebounded to levels 40% below the preinjection baseline. In contrast to low-dose heroin, this tonic decrease had a longer duration, exceeding 2–3 h. Interestingly, oxygen levels rapidly returned to baseline levels when the rat initiated locomotor activity following profound drug-induced hypoactivity.

Heroin-induced increases in NAc glucose were also greatly enhanced following the large-dose drug injection (Fig. 6*B*). The increase was four times greater in both magnitude and duration; the concentration of glucose doubled with respect to its absolute baseline. As seen following administration of the 200 μg/kg heroin dose, the elevation in glucose was preceded by a transient glucose decline immediately following the large dose heroin injection. The decrease observed following administration of the 3.2 mg/kg dose, however, was much larger in magnitude and longer in duration.

Heroin at the 3.2 mg/kg dose also induced different temperature responses, with the appearance of strong decreases in NAc and muscle temperature (∼1.5–2.0°C), which were evident for 3–4 h after injection when the rats were fully sedated and immobile (Fig. 6*C*). Then, the temperatures rebounded to levels well above (∼2°C) the preinjection baseline, slowly decreasing thereafter.

Of the six rats tested with the large heroin dose, one animal died during the recording session (Fig. 6*A*). In this case, we observed an immediate sharp drop in oxygen (80% decrease) followed by a more tonic and prolonged oxygen decrease (40% decrease) and a robust terminal oxygen drop associated with cessation of breathing.

## Discussion

We used high-speed amperometry in awake, freely-moving rats to examine the effects of heroin on extracellular levels of oxygen and glucose in the NAc, the relationships between these two substances, and the mechanisms underlying the change in their brain levels after heroin exposure. We also assessed whether heroin-induced changes in NAc oxygen and glucose are related to heroin-induced changes in temperature with a focus on intra-brain heat production, a reliable measure of metabolic brain activity.

### Heroin-induced hypoxia followed by hyperglycemia

We found that heroin at a low dose [100 μg/kg, within the dose range of rat self-administration (25–200 μg/kg; Gerber and Wise, 1989) and human drug consumption (https://www.erowid.org/)] caused a rapid, strong, and transient drop in NAc oxygen. The oxygen drop increased in magnitude and duration when the dose was doubled to 200 μg/kg. Respiratory depression was seen after heroin injection and the decrease in blood oxygen levels is likely the cause of the brain hypoxic response. This gradient-dependent mechanism was confirmed by our oxygen recordings in the subcutaneous space. In contrast to other tissues, which continuously consume oxygen, the subcutaneous location contains minimal or no metabolic activity and oxygen decreases in this area result from decreases in arterial oxygen levels. The decrease in oxygen in the subcutaneous space was larger and more prolonged than that in the NAc, despite the peripheral vasoconstriction indicated by our temperature recordings. Therefore, the decrease in NAc oxygen likely results from respiratory depression, a fall in blood oxygen levels, and diminished gradient-dependent entry of oxygen from the arterial blood into the brain. A global decrease in fMRI signal, suggesting decreased blood oxygenation, has been found in anesthetized rats that received intravenous heroin at the same dose (Xu et al., 2000); decreased oxygenation disappeared when the rats were placed under artificial respiration.

Heroin and other opioids cause respiratory depression (Jaffe, 1990; Pattinson, 2008), and there are many studies on the mechanisms of opioid-induced respiratory depression (Lalley, 2008; Stucke et al., 2008; Lalley et al., 2014). However, it is difficult to study respiration in awake rodents (Kabir et al., 2010) and opioid-induced respiratory depression involves changes in multiple interrelated parameters, including breathing rate, tidal volume, impaired pulmonary gas exchange, and blunting of respiratory responsiveness to hypoxia and hypercapnia (Lalley et al., 2014). Our approach, direct oxygen monitoring in brain and subcutaneous space, allows us to quantify the outcome of respiratory depression and assess actual changes in brain oxygen levels, which has not been done before in freely-moving rats.

We also showed that heroin induced strong brain hyperglycemia. This effect was slower but more prolonged than the decrease in oxygen levels. The glucose increase could be the result of one of two factors: a rise in blood glucose levels and/or local vasodilation. Using data obtained by glucose monitoring in the subcutaneous space, we can reject the contribution of the first factor. Heroin rapidly decreased skin glucose levels; this decrease is caused by skin vasoconstriction, which our thermorecording experiments suggest occurs during the period of glucose increase following the heroin injection. We also confirmed that the subcutaneous space is sensitive to changes in blood glucose levels, showing rapid and strong increases after intravenous injection of glucose. Therefore, intravenous heroin induces distinct changes in glucose, increasing its brain levels and decreasing its levels in peripheral tissues. As such, NAc hyperglycemia likely results from local vasodilation.

NAc oxygen levels displayed the most rapid dynamics of any physiological parameter in our study, decreasing significantly before any increases in glucose. The glucose rise began when oxygen levels were at their nadir and the changes in the two substances were tightly correlated for the next 6–7 min. Therefore, the rise in glucose levels appears to be dependent on and secondary to the hypoxia due to heroin-induced respiratory depression. Respiratory depression, in addition to decreasing brain oxygen levels, causes increased brain CO_2_ levels, which in turn causes vasodilation (Schmidt and Kety, 1947; Kontos, 1981; Battisti-Charbonney et al., 2011). Therefore, the heroin-induced rise in brain CO_2_ levels likely causes cerebral vessels to dilate, leading to increased cerebral blood flow and increased glucose entry into the brain.

### Relationship between heroin-induced temperature changes, metabolic activation, and NAc oxygen and glucose

Energy used for brain metabolism is transformed into heat (Siesjö, 1978). Therefore, heat production is the ultimate consequence of brain metabolic activity. As shown in our study, low dose intravenous heroin (100 μg/kg) moderately increases brain temperature. By calculating skin-muscle temperature differentials, we showed that this temperature increase results primarily from diminished heat dissipation due to peripheral vasoconstriction. As suggested by changes in the NAc-muscle temperature differential, heroin also induces brain metabolic activation. This effect, however, is relatively weak and appears 10–30 min after injection, following major changes in both oxygen and glucose. After heroin injection at a 200 μg/kg dose, we observed a minor postinjection decrease in NAc temperature that preceded a stronger and more prolonged temperature elevation. This effect precedes stronger and delayed vasoconstriction that likely mediates the subsequent temperature elevation. As shown by analyzing temperature differentials, this initial decrease in temperature results not from metabolic inhibition but from transient skin vasodilation that increases heat dissipation to the external environment. The lack of correlation between metabolic brain activation and two critical metabolism-dependent parameters is inconsistent with the classic “homeostatic” dogma that oxygen and glucose enter the brain during and after metabolic activation to compensate for possible deficits due to enhanced utilization of oxygen and glucose (Siesjö, 1978; Sokoloff, 1999; Mergenthaler et al., 2013). Rather, it appears that a rapid decline in brain oxygen after heroin injection is due to respiratory depression that occurs before any detectable changes in brain metabolism. Similarly, the rise in glucose results not from metabolic deficit but from brain hypoxia and a parallel rise in CO_2_ that triggers local vasodilation and enhances glucose entry into the brain. Therefore, it appears that heroin-induced changes in oxygen and glucose are not directly related to drug-induced changes in metabolic activity. In contrast to rapid changes in oxygen and glucose, intra-brain heat production, a measure of metabolic activation, began to increase slowly ∼10 min after heroin injection when the rats initiate locomotion after postinjection motor inactivity.

### Transformation of NAc oxygen and glucose responses with increased heroin dose

Our study focused primarily on the effects of low to moderate self-administering heroin doses, but we also examined the effect of a very high heroin dose on brain oxygen and glucose to determine the effect of heroin “overdose” on these physiological parameters. When heroin was injected at the 200 μg/kg dose, the observed oxygen decrease and subsequent glucose rise were clearly enhanced compared with those induced by the 100 μg/kg dose. While this dose-dependent progression was expected, we observed an unanticipated slight, transient glucose drop immediately after heroin injection (30–180 s) before the subsequent enhanced glucose rise. The mechanisms underlying this transient drop in glucose are unclear, but could be related to stronger hypoxia and hypercapnia in both the brain and periphery that cause peripheral vasodilation. In contrast to the monophasic decrease in the skin-muscle differential seen with a 100 μg/kg dose, the change became biphasic with 200 μg/kg, suggesting transient postinjection skin vasodilation followed by stronger and prolonged vasoconstriction. This change appears responsible for the initial drop in brain and muscle temperatures after heroin injection at a 200 μg/kg dose.

These changes in oxygen and glucose responses showed a robust progression when rats were injected with a very high heroin dose (3.2 mg/kg) that is well above the dose range of heroin self-administration. In this case, NAc oxygen levels dropped rapidly and strongly after injection but then slightly recovered and remained at low levels for a long time. The rise in glucose was also larger in magnitude and longer in duration, but was preceded by a stronger drop for the first several minutes after drug injections. These changes in brain oxygen and glucose were coupled with a robust and prolonged decrease in brain and muscle temperature and deep sedation. We found that the large heroin dose decreased brain oxygen to pathological levels, resulting in the death of one rat, which highlights the relevance of these data for understanding the physiological mechanisms of heroin overdose.

### Conclusions and functional implications

Our study demonstrates a dynamic interaction between heroin-induced brain hypoxia and resultant hyperglycemia and clarifies the role of respiratory depression, peripheral vasoconstriction, and local vasodilation in mediating these central effects. We also show that heroin-induced changes in brain oxygen and glucose are rapid and appear to be independent of subsequent slower changes in brain metabolic activity. Because our data were collected in freely-moving rats using high temporal resolution analyses and human-relevant doses and routes of drug administration, they likely represent the physiological dynamics of two critical metabolic parameters that occur in humans during initial exposure to heroin and under some overdose conditions. A caveat in our study, however, is that the translation of experimental data from rats to humans, particularly regarding dose “equivalency,” is not straightforward for many reasons, including known differences in basic metabolism (Schmidt-Nielsen, 1997) and possible, still unstudied species differences in pharmacokinetics of heroin and its active metabolites in the brain.

## Synthesis

Reviewing Editor: Quentin Pittman, University of Calgary Decisions are customarily a result of the Reviewing Editor and the peer reviewers coming together and discussing their recommendations until a consensus is reached. When revisions are invited, a fact-based synthesis statement explaining their decision and outlining what is needed to prepare a revision will be listed below. The following reviewer(s) agreed to reveal their identity: Aldo Badiani, George Rebec

Both reviewers, who were the reviewers of your first submission to JNS are in agreement that your manuscript is now acceptable for publication. However, one reviewer has the following comment:

“I have just one “minor” request concerning the interpretation of heroin-induced decrease in NAc temperature. Since the authors argue that this is an index of local metabolism, it would be appropriate to cite the report by Kiyaktin (1994) that i.v. infusions of heroin decrease DA-dependent electrochemical signal (Fig. 2).”

Please deal with this in your resubmission by either incorporating the statement and reference or alternately provide a rebuttal indicating why you do not wish to revise it as suggested. I will make a decision without further external review.
